# Hemodialysis-Associated Immune Dysregulation in SARS-CoV-2-Infected End-Stage Renal Disease Patients

**DOI:** 10.3390/ijms24021712

**Published:** 2023-01-15

**Authors:** Cecilia González-Cuadrado, Paula Jara Caro-Espada, Marta Chivite-Lacaba, Alberto Utrero-Rico, Claudia Lozano-Yuste, Elena Gutierrez-Solis, Enrique Morales, Justo Sandino-Pérez, Francisco Javier Gil-Etayo, Luis Allende-Martínez, Rocio Laguna-Goya, Estela Paz-Artal

**Affiliations:** 1 Instituto de Investigación Sanitaria 12 de Octubre (imas12), 28041 Madrid, Spain; 2 Department of Nephrology, Hospital Universitario 12 de Octubre, 28041 Madrid, Spain; 3 Department of Medicine, Facultad de Medicina, Universidad Complutense de Madrid, 28040 Madrid, Spain; 4 Department of Immunology, Hospital Universitario 12 de Octubre, 28009 Madrid, Spain; 5 Centro de Investigación Biomédica en Red de Enfermedades Infecciosas (CIBERINFEC), Instituto de Salud Carlos III, 28029 Madrid, Spain; 6 Department of Immunology, Ophthalmology and ENT, Facultad de Medicina, Universidad Complutense de Madrid, 28040 Madrid, Spain

**Keywords:** hemodialysis, SARS-CoV-2 infection, COVID-19, immune dysregulation

## Abstract

Patients on hemodialysis show dysregulated immunity, basal hyperinflammation and a marked vulnerability to COVID-19. We evaluated the immune profile in COVID-19 hemodialysis patients and the changes associated with clinical deterioration after the hemodialysis session. Recruited patients included eight hemodialysis subjects with active, PCR-confirmed SARS-CoV-2 infection, five uninfected hemodialysis patients and five healthy controls. In SARS-CoV-2-infected hemodialysis patients TNF-α, IL-6 and IL-8 were particularly increased. Lymphopenia was mostly due to reduction in CD4+ T, B and central memory CD8+ T cells. There was a predominance of classical and intermediate monocytes with reduced HLA-DR expression and enhanced production of pro-inflammatory molecules. Immune parameters were analysed pre- and post-hemodialysis in three patients with COVID-19 symptoms worsening after the hemodialysis session. There was a higher than 2.5-fold increase in GM-CSF, IFN-γ, IL-1β, IL-2, IL-6, IL-17A and IL-21 in serum, and augmentation of monocytes-derived TNF-α, IL-1β and IL-8 and CXCL10 (*p* < 0.05). In conclusion, COVID-19 in hemodialysis patients associates with alteration of lymphocyte subsets, increasing of pro-inflammatory cytokines and monocyte activation. The observed worsening during the hemodialysis session in some patients was accompanied by augmentation of particular inflammatory cytokines, which might suggest biomarkers and therapeutic targets to prevent or mitigate the hemodialysis-related deterioration during SARS-CoV-2 infection.

## 1. Introduction

Patients with end-stage renal disease (ESRD) on renal replacement therapy by hemodialysis (HD) are particularly vulnerable to coronavirus disease 2019 (COVID-19). They are at increased risk of infection (2.9% of incidence) because their need for regular dialysis makes social isolation more difficult [1]. Their risk of developing severe COVID-19 is also higher, due to their high average age and multiple co-morbidities including hypertension, diabetes and obesity [2]. Studies reveal that long-term HD patients have higher rates of intensive care unit (ICU) admissions, and their mortality exceeds 20% in both Europe and the United States [1,3,4,5,6], being superior to other risk groups such as renal transplant patients [7].

Long-term HD patients exhibit a dysregulation of the adaptive and innate immune system, which can also render them more susceptible to COVID-19 [8,9,10]. ESRD patients receiving chronic HD show premature immunosenescence, with decreased CD4+ and CD8+ T and B cells [11,12]. Lymphocyte function is impaired, showing increased susceptibility to activation-induced apoptosis [13,14,15]. In addition, the constant contact with uremic toxins and non-self-antigens from HD biomaterials also leads to chronic inflammation [16]. Maintenance HD patients have a higher proportion of inflammation-related monocytes with a pre-activation phenotype [17] which, together with neutrophils, overproduce pro-inflammatory cytokines like interleukin (IL)-1β, IL-6, IL-8, tumour necrosis factor-alpha (TNF-α), monocyte chelator protein-1 (MCP-1), and interferon gamma (IFN-γ) [18,19,20]. Enhanced neutrophil apoptosis accompanying myeloperoxidase release during HD has been also observed [21], all of which contribute to the inflammatory state in HD patients.

SARS-CoV-2 infection can also result in a deep alteration of the immune profile and, consequently, a severe course of COVID-19 in high-risk patients. It has been suggested that COVID-19 immunopathology is provided both by overreactive innate immune response and an exhausted T cell response [22]. SARS-CoV-2 may cause hyperinflammation with increased cytokine and chemokine production and high levels of inflammatory monocytes and macrophages [23,24,25,26].

The interplay between SARS-CoV-2 and the immune system of chronic HD patients is not well understood yet. Alterations in the immunity associated with premature re aging in ESRD may contribute to severity of COVID-19 [27]. It has been described that chronic HD patients with SARS-CoV-2 infection display higher plasma pro-inflammatory cytokine levels than uninfected HD patients. This increase in cytokine levels correlates with the severity of COVID-19 [28]. Furthermore, some studies reveal that HD patients make delayed and attenuated responses towards mRNA COVID-19 vaccines which tend to rapidly decline over time. These factors, together with the emergence of the new variants of concern (VOCs) may make HD patients particularly vulnerable to severe courses of COVID-19 [29].

We observed that some chronic HD patients suffering acute COVID-19 presented worsening of symptoms, with recurrence of fever, dyspnea, and general malaise, after HD sessions. We hypothesized that ESRD patients receiving regular HD present an augmented baseline inflammatory state that would increase during acute COVID-19. The exacerbation of symptoms after HD would be accompanied by a further dysregulation of the innate immunity with augmented inflammation. This study has aimed to evaluate the immune profile associated with COVID-19 in HD patients and the immune profile changes associated with worsening after the HD session.

## 2. Results

### 2.1. Long-Term HD Patient Characteristics

We recruited eight chronic HD patients who were diagnosed with COVID-19 by RT-PCR and five chronic HD patients without evidence of SARS-CoV-2 infection, all of them in HD treatment. No significant differences were observed in the age and sex distribution between the HD and HD + COVID-19 patient groups (Table 1). There were no differences in comorbidities or time in HD between both groups. HD + COVID-19 patients showed higher inflammation, measured as higher levels of C-reactive protein and IL-6, and lower lymphocytes and higher aspartate aminotransferase in routine clinical tests on the sampling day compared to uninfected HD patients. Two HD + COVID-19 patients died. Samples from HD + COVID-19 patients were obtained during the acute phase of disease. The median time from symptoms onset (post-symptom onset, PSO) to sample collection was 11 days (IQR 6-14). Detailed data of each HD + COVID-19 patient can be found in (Table S1).

### 2.2. Long-Term HD Patients Showed a Pro-Inflammatory Serum Cytokine Profile Which Increased with COVID-19

**Figure 1:**
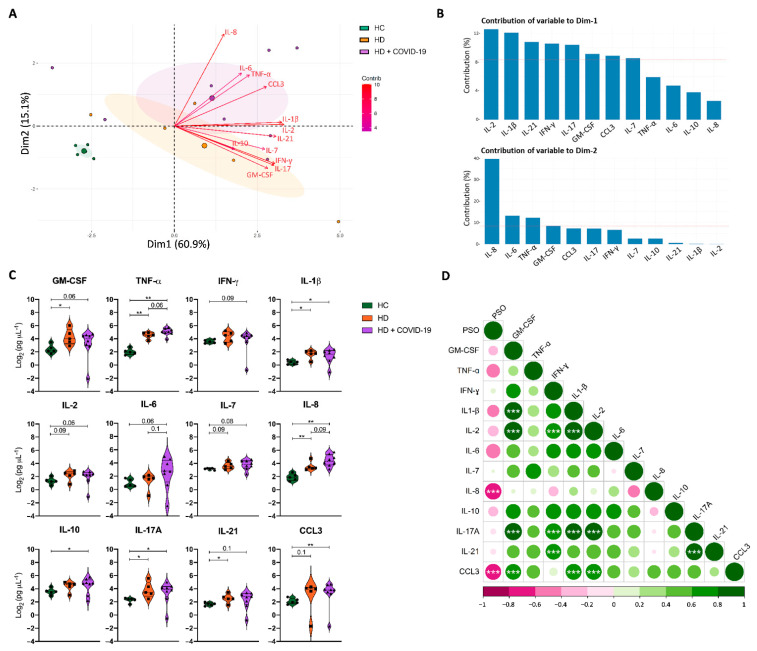
Differential cytokine profile in HC, HD and HD + COVID-19 patients. (**A**) Principal component analysis (PCA) of 12 cytokines in plasma from HC, HD and HD + COVID-19 patients shows an inflammatory cytokine profile in HD patients, further increased by SARS-CoV-2 infection. Each small dot represents a sample; large dots represent the median of the group and shaded area is the 95% confidence interval. (**B**) Contribution of each cytokine to PCA dimensions 1 and 2. Red dashed line represents the mean of all cytokine contributions. (**C**) Levels of 12 cytokines in plasma from HC, HD and HD + COVID-19 patients. (**D**) Correlation analysis between time of sampling relative to symptom onset and plasma cytokine levels in HD + COVID-19 patients. Positive correlations appear in green, and negative correlations appear in pink. The size and the colour gradient of the circle correspond to the magnitude of the correlation. Linear regressions were performed using Spearman’s rank test, *, *p* < 0.05; **, *p* < 0.01; ***, *p* < 0.001. PSO: Days post-symptom onset.

We evaluated the inflammatory state of chronic HD patients with and without SARS-CoV-2 infection according to the expression of 12 serum cytokines and compared it to healthy controls (HC). The principal component analysis (PCA) separated the three cohorts into discrete clusters and revealed that ESRD patients on chronic HD had a basal pro-inflammatory profile, in comparison to HC, which was further increased by SARS-CoV-2 infection (Figure 1A). The cytokines that differed the most between HC and HD patients belonged to dimension 1, in which among all cytokines contributing over the mean (Figure 1B), GM-CSF, IL-1β, IL-21, IL-17A, were significantly increased in HD patients (Figure 1C). The cytokines grouped in dimension 2 discriminated infected HD from uninfected HD patients. Of those, IL-8, IL-6 and TNF-α were the most enhanced in HD + COVID-19 patients (Figure 1B,C).

In HD + COVID-19 patients serum cytokine levels correlated negatively with PSO days, reaching statistical significance in the case of the neutrophil chemotactic factor IL-8 and the macrophage inflammatory protein-1α CCL3 (Figure 1D). This suggests that SARS-CoV-2 causes an increase in pro-inflammatory cytokines in chronic HD patients in the beginning of the infection, which then wanes. In addition, significant direct correlations were observed among GM-CSF, IL-1β, IL-2, IL-17 and CCL3. Of note, IL-8 tended to inversely correlate with IL-7, IFN-γ and IL-10, showing the concurrence between resolution of the inflammatory state, with decrease in some pro-inflammatory cytokines such as IL8, increase in cytokines associated with lymphopenia recovery and effective immune responses, such as IL-7 and IFN- γ, respectively, and production of the anti-inflammatory cytokine IL-10.

### 2.3. Effect of COVID-19 on Lymphocyte and Monocyte Phenotype and Function in Chronic HD Patients

**Figure 2:**
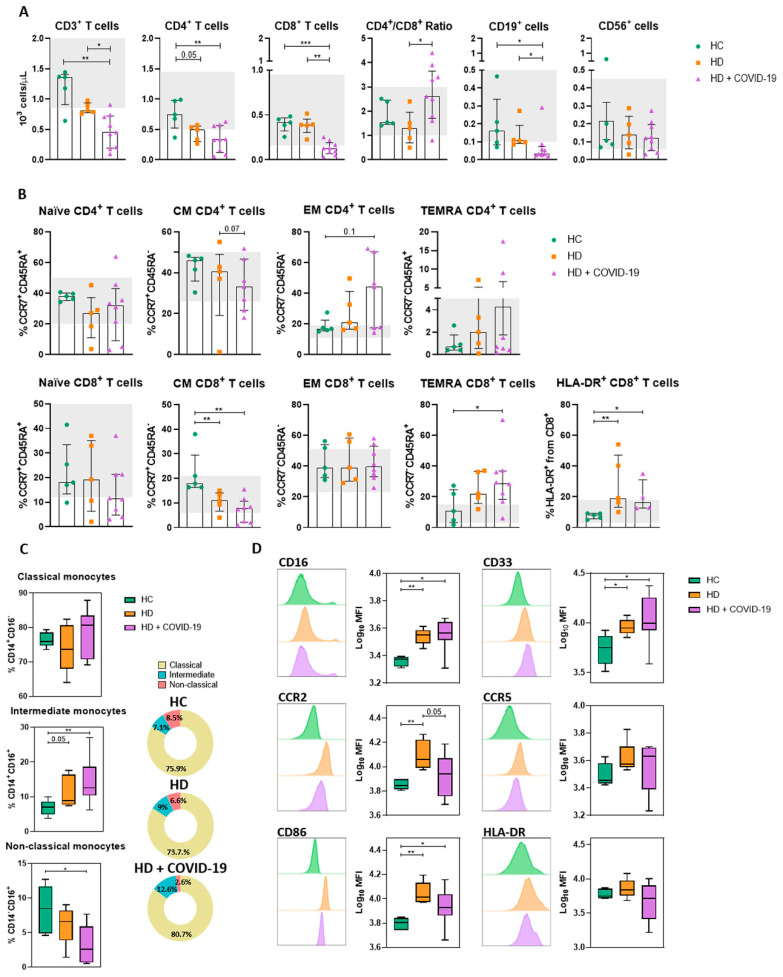
Flow cytometry analysis of adaptive and innate cell subpopulations of HC, HD and HD + COVID-19 patients. (**A**) T, B and NK cell phenotype. (**B**) Percentage of CD4+ and CD8+ T cell subsets (naïve, central memory [CM], effector memory [EM] and terminally differentiated effector memory [TEMRA]) according to the expression of CD45RA and CCR7. Grey regions depict reference frequency ranges obtained from representative healthy donors in our clinical immunology laboratory. (**C**) Comparison of classical (CD14+CD16-), intermediate (CD14+CD16+) and non-classical (CD14-CD16+) monocytes between the three cohorts. (**D**) Examples and comparison of expression of different surface markers (CD16, CD33, CCR2, CCR5, CD86 and HLA-DR) on CD14+ monocytes from HC, HD and HD + COVID-19 patients, *, *p* < 0.05; **, *p* < 0.01; ***, *p* < 0.001.

We next analysed T, B and NK cell populations in HD and HD + COVID-19 patients and compared them to HC. We found a mild lymphopenia (<1.2 × 10^3^ lymphocytes per µL) in non-infected HD patients compared to HC, mainly due to reduced helper CD4+ T (Th) cell counts (Figure 2A). However, HD + COVID-19 patients showed the largest reduction in total lymphocyte numbers, which affected all lymphocyte populations studied and was especially marked in cytotoxic CD8+ T (Tc) cells, resulting in an increased Th/Tc coefficient (Figure 2A). Within the reduced CD4+ T cell compartment, HD + COVID-19 patients had a tendency towards decreased frequencies of central memory (CM) CD4+ T cells and increased effector memory (EM) and terminally differentiated effector memory (TEMRA) CD4+ T cells compared to HC and uninfected HD patients (Figure 2B). Regarding CD8+ T cell distribution, a slight reduction in naïve CD8+ T cells was observed in HD + COVID-19 patients compared with HC and HD patients. In the memory CD8+ compartment we observed a significant reduction in CM CD8+ T cells in HD patients compared to HC that was further aggravated in HD + COVID-19 patients, as well as a significant increase in TEMRA and CD8+ T cells positive for the activation marker human leukocyte antigen-DR (HLA-DR) in both HD and HD + COVID-19 patients compared to HC (Figure 2B). There was no clear correlation between PSO days and T, B and NK subpopulations (Figure S2A).

Monocyte count was within normal range (0.2–0.9 × 10^3^ cells/µL) in all patients (Table 1). We evaluated the effect of SARS-CoV-2 infection on monocyte subpopulations and expression of chemotactic, co-stimulatory and antigen-presenting molecules on the surface of monocytes, as well as their cytokine production after in vitro overnight stimulation. There was an increase in intermediate monocytes and a decrease in non-classical monocytes in HD patients, which was further accentuated in HD + COVID-19 patients (Figure 2C). CD14+ monocytes phenotype was analysed by surface expression of CD86, a co-stimulatory receptor, HLA-DR, an MHC-class II receptor that supports antigenic presentation to CD4+ lymphocytes, and CCR2 and CCR5 receptors involved in chemotaxis. We found that monocytes of chronic HD patients with or without infection displayed higher expression of co-stimulation and migration molecules than HC. A trend toward down-regulation of CD86 and HLA-DR was observed in HD + COVID-19 patients (Figure 2D).

**Figure 3:**
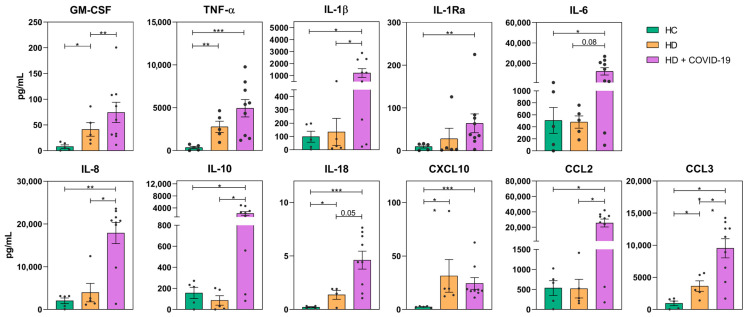
Analysis of monocyte function in HC, HD and HD + COVID-19 patients. Monocyte production of 11 cytokines measured in supernatant of monocyte in vitro culture upon LPS stimulation, *, *p* < 0.05; **, *p* < 0.01; ***, *p* < 0.001.

We next evaluated the capacity of monocytes to secrete cytokines upon in vitro LPS stimulation. In comparison to HC, monocytes from chronic HD patients secreted significantly higher amounts of pro-inflammatory cytokines such as GM-CSF, TNF-α, IL-18, CXCL10 and CCL3, suggesting that these monocytes had a higher basal activation state (Figure 3). Monocytes of HD + COVID-19 patients secreted significant higher levels of the pro-inflammatory cytokines GM-CSF, IL-1β, IL8, IL-10, CCL2 and CCL3 compared to non-infected HD patients. Levels of TNF-α, IL-1Ra, IL-6 and IL-18 were also higher in HD + COVID-19 than in HD patients, without reaching statistical significance. Time PSO negatively correlated with the amount of these cytokines secreted by LPS-stimulated monocytes from infected HD patients, reaching statistical significance for CXCL10 and CCL3. This suggested a normalization of the hyperactivation state of circulating monocytes over the course of SARS-CoV-2 infection (Figure S2B).

### 2.4. Increased Inflammatory Status and Capacity of Monocytes to Secrete Cytokines in Patients Who Worsen Their COVID-19 Symptoms after HD Sessions

**Figure 4:**
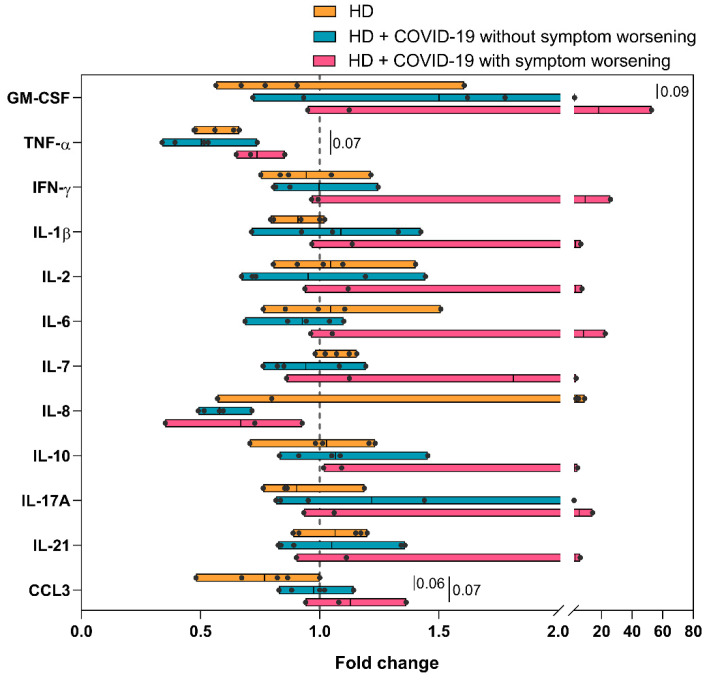
Change in serum cytokine levels after hemodialysis. Comparison of fold change in cytokine levels pre- and post-hemodialysis between uninfected HD patients, HD + COVID-19 patients without worsening of COVID-19 symptoms and HD + COVID-19 patients with worsening of COVID-19 symptoms after receiving hemodialysis. Dotted line represents unchanged cytokine level.

We observed that three out of the eight HD + COVID-19 patients studied experienced a poor tolerance to HD sessions with increased dyspnea, fever, myalgia, and general malaise after the HD session (patients 1*, 5 and 7, Table S1). To test if this worsening of COVID-19 symptoms could be immune-mediated, immunological parameters were measured before and after the HD session and the differences were expressed as fold change. We found no major differences in serum cytokine levels between pre- and post-dialysis values in both HD patients and HD patients without symptom worsening (Figure 4). However, HD patients with COVID-19 symptom worsening showed up-regulation (more than 2.5-fold increase) of GM-CSF, IFN-γ, IL-1β, IL-2, IL-6, IL-17A and IL-21, and down-regulation of TNF-α and IL-8 serum levels after the dialysis procedure (Figure 4).

**Figure 5:**
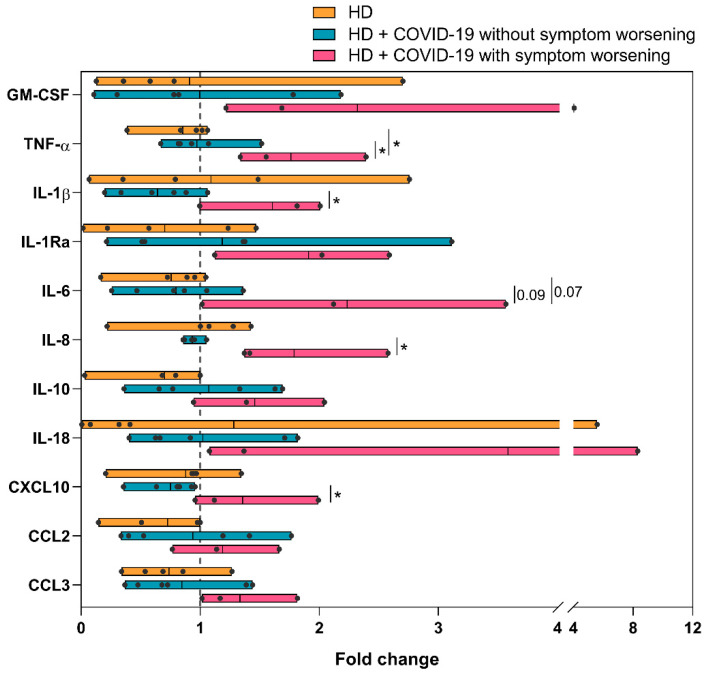
Change in the in vitro cytokine production by monocytes after hemodialysis. Comparison of fold change in cytokines levels pre- and post-hemodialysis between uninfected HD patients, HD + COVID-19 patients without worsening of COVID-19 symptoms and HD + COVID-19 patients with worsening of COVID-19 symptoms after receiving hemodialysis. Dotted line represents unchanged cytokine level, *, *p* < 0.05.

HD session did not significantly modify the distribution of lymphocyte or monocyte subsets in long-term HD patients with worsening of COVID-19 symptoms, except for an increase in TEMRA CD8+ cells and inferior recovery of CM CD8+ T cells in comparison with uninfected or infected patients who did not worsen after HD (Figure S3). Notably, the measurement of cytokines in supernatants from LPS-stimulated monocytes isolated before and after HD showed that the capacity of monocytes to secrete cytokines was enhanced by the HD session in patients with worsening of symptoms, with increased (>1.5-fold) release of GM-CSF, TNF-α, IL-1β, IL-1Ra, IL-6, IL-8, and IL-18 post-HD. This increase did not occur in uninfected or infected HD patients without clinical deterioration. The augmentation of cytokines after HD in worsening vs. non-worsening patients reached statistical significance for TNF-α, IL-1β and IL-8 and CXCL10 (Figure 5).

## 3. Discussion

Several studies have reported that ESRD patients on chronic dialysis exhibit compromised immunity with high susceptibility to infections, which is a major cause of death among these patients [8,10,30]. In addition, the accumulation of toxins due to renal failure as well as the dialysis procedure can lead to hyperactivation of the innate immunity and suboptimal adaptive immune responses [9,11,16,30]. Accordingly, long-term HD patients may have abnormal responses to SARS-CoV-2 virus, leading to severe COVID-19 with worse clinical outcomes than the general population [27].

To understand the immune response triggered by SARS-CoV-2 in chronic HD patients we evaluated markers from both innate and adaptive immunity. First, we studied the profile of different soluble pro-inflammatory cytokines (GM-CSF, TNF-α, IFN-γ, IL-1β, IL-2, IL-6, IL-7, IL-10, IL-17A, IL-21) and chemokines mediating monocyte and neutrophil recruitment (IL8, CCL3), whose exacerbated production is associated with severe COVID-19 [23,26,31,32]. In keeping with previous studies [28,33] we observed an increase in proinflammatory cytokines in HD patients, which was more pronounced during SARS-CoV-2 infection (Figure 1). Interestingly, levels of pro-inflammatory cytokines reduced as time passed from the beginning of infection, while IL-7 and IFN-γ and IL-10 tended to increase, showing the recovery of a more functional immune system and of mechanisms to counterbalance inflammation.

Regarding adaptive immunity, long-term HD patients presented a reduction in the frequency of total helper T and B lymphocytes, and an increase in terminally differentiated effector helper and cytotoxic T lymphocytes, as well as activated HLA-DR+ CD8+ T lymphocytes, compared to HC (Figure 2). These disturbances are consistent with the lymphocyte depletion and lymphocyte subset alterations observed in chronic renal failure [11,12,14,34], and were further accentuated by SARS-CoV-2 infection. These changes closely resemble previous observations in which lymphopenia has been established as a strong predictor of severe COVID-19 risk in the general population [23,35] as well as in COVID-19 HD patients [36]. In addition, the disbalance between the relatively low CM T cells and the augmented EM and TEMRA compartments in HD + COVID-19 patients could prevent an optimal response against SARS-CoV-2 and contribute to the increased COVID-19 severity in HD patients [37].

Monocytes and macrophages have been reported as two of the main cell types involved in the exacerbation of the immune response in patients with COVID-19 [26,38,39]. As previously reported [17,40], our observations showed that chronic HD patients present increased intermediate monocytes in comparison to HC and a trend towards diminished non-classical monocytes with high expression of CD86, CCR2, CD16 and CD33. These modifications of the monocyte compartment profile in HD patients could be related to the chronic uremic state together with the frequent HD sessions [16,17,18,19,41]. We confirmed that SARS-CoV-2 infection in HD patients further amplified the increase in intermediate and classical monocytes and the decrease in non-classical monocytes. These modifications in monocyte subsets have been associated with more inflammation and M1-polarization of the immune response [42], and worse COVID-19 outcome in the general population [26,43,44]. The tendency in monocytes from infected HD patients to reduce their CD86 and HLA-DR expression, which reduces their antigen presentation and co-stimulatory capacity, has been associated with severe COVID-19 [26,38,45] and could also partly explain the increased risk of severe outcome in chronic HD patients with COVID-19.

When we evaluated the function of monocytes in vitro, we found that production of all pro- and anti-inflammatory cytokines analysed was higher in HD patients compared to HC (Figure 3), probably as an expression of the inflammation state in renal patients [9,46]. Therefore, our results support that HD has a probable effect in increasing monocytes with an inflammatory phenotype and increased cytokine secretory capacity. Monocytes from HD + COVID-19 patients showed an enhanced capacity for pro-inflammatory cytokine production, with GM-CSF, IL-1β, IL-8, IL-10, CCL2 and CCL3 as the most increased cytokines compared with uninfected HD patients. This vast increase in cytokine levels in patients with a highly immunoreactive baseline state may contribute to the overreaction triggered by SARS-CoV-2 infection in long-term HD patients [18,19,43].

To our knowledge, this is first study to analyse in depth changes in both innate and adaptive immune profiles of chronic HD patients with COVID-19 before and after HD session. These results are limited by the low statistical power due to the small number of patients per group when we divided them depending on whether their symptoms worsened after HD session. Nevertheless, we found that the HD patients who display worsening of COVID-19 symptoms exhibited a large increase in serum cytokine levels after HD session (>2.5-fold increase for GM-CSF, IFN-γ, IL-1β, IL-2, IL-6, IL-17A and IL-21), which was not observed in uninfected HD patients or COVID-19 HD patients without clinical deterioration. In addition, monocytes from patients with clinical deterioration after HD session had increased production of GM-CSF, TNF-α, IL-1β, IL-1Ra, IL-6, IL-8 and IL-18 which supports reports showing that dialysis sessions can pre-activate monocytes in HD patients [16,18,19,47]. This increase in circulating pro-inflammatory cytokines and in the capacity to release cytokines by monocytes during the dialysis session could possibly explain the appearance or worsening of fever, myalgia, and other COVID-19 related symptoms in some patients.

Another limitation of our study is that all patients included in the different cohorts were recruited from the first pandemic wave. They were all infected by the original Wuhan SARS-CoV-2 strain, and it would be relevant to show if other strains, namely Omicron, cause similar immune alterations in chronic HD patients. Omicron has been reported to cause less severe infection than Delta or other previous SARS-CoV-2 strains in HD patients [48]. Moreover, most HD patients are currently vaccinated. It would be interesting to study the immune dysregulation caused by SARS-CoV-2 infection and the COVID-19 severity in these vaccinated HD patients in relation to their response to vaccination.

In summary, our results support that chronic HD associates with lymphopenia, immunosenescence and an inflammatory state. These innate and adaptive immune alterations are further amplified by SARS-CoV-2 infection and could partly contribute to the increased COVID-19 severity in the HD patient population. Furthermore, we report a relationship among worsening of COVID-19 symptoms immediately after a single dialysis session, monocyte activation and augmentation of pro-inflammatory cytokine levels during the dialysis process. Our results suggest potential therapeutic targets to prevent or mitigate the HD-related deterioration during SARS-CoV-2 infection.

## 4. Materials and Methods

### 4.1. Patients and Controls

Thirteen ESRD subjects on HD were recruited at Hospital Universitario 12 de Octubre (Madrid, Spain) between 27 April and 29 September 2020: 8 patients had active, PCR-confirmed SARS-CoV-2 infection at the time of the study (HD + COVID-19) and 5 were uninfected (HD). One HD + COVID-19 patient (P1, Table S1) was analysed independently at two time points of COVID-19 evolution. Blood samples were collected before and after HD session. Four out of five uninfected HD patients received high-volume online hemodiafiltration, and one patient was treated with expanded HD. All HD + COVID-19 patients were treated with high-flux dialyzers. The average of ultrafiltration volume was 2.1 L/session. Anticoagulation was performed by discontinuous heparin administration. Five age- and sex-matched healthy controls were also analysed. All participants signed an informed consent. The study was approved by the institutional review board (reference no. 20/167).

### 4.2. Analysis of Lymphocyte and Monocyte Subsets by Flow Cytometry

T cell subsets were evaluated in whole blood and monocyte subsets were analysed in PBMC (see antibodies used for staining in Table S2). Samples were acquired with FACSCanto II (BD Bioscience) or Navios flow cytometers (Beckman Coulter, San Jose, CA, USA) and analysed using FlowJo v10.6.2 (Treestar Inc., Woodburn, OR, USA) or Kaluza Software (Beckman Coulter). Gating strategy is shown in Figure S1.

### 4.3. Monocyte In Vitro Cultures

For ex vivo stimulation and cytokine production, monocytes were isolated from PBMC (EasySep human monocyte enrichment without CD16 depletion kit; StemCell, Vancouver, Canada), following manufacturer’s instructions. Purified monocyte fraction was plated in 96-well flat bottom plate (105 monocytes/well) and incubated for 1 h in RPMI1640 supplemented with 10% fetal bovine serum (FBS), 1% penicillin-streptomycin, 1% L-glutamine and 1% sodium-pyruvate at 37 °C. After that, cells were washed with warmed PBS and fresh culture medium was added with 10 ng/mL of LPS. Supernatant was collected after overnight incubation and stored at −20 °C.

### 4.4. Multiplex Detection of Cytokines

In plasma samples, 12 cytokines were detected using the multiplex Human Cytokine/growth high sensitivity panel: GM-CSF, TNF-α, IFN-γ, IL-1β, IL-2, IL-6, IL-7, IL-8, IL-10, IL-17A, IL-21 MIP-1a (CCL3) detection kit, and in supernatant samples, 11 cytokines were detected using the Human Cytokine/growth factor panel: GM-CSF, TNF-α, IL-1β, IL-1Ra, IL-6, IL-8, IL-10, IL-18, CXCL10, MCP-1 (CCL2) and MIP-1a (CCL3) kit (both from Millipore). Plates were analysed by Luminex 100 (One Lambda, Waltham, MA, USA).

### 4.5. Statistics

Continuous numeric variables are shown as median with interquartile range (IQR). Non-parametric Mann–Whitney U was applied for comparison within all groups. Categorical variables are represented as N and percentage and were compared by using Fisher exact or Chi-square test when appropriate. Principal component analysis (PCA) of log-transformed cytokine levels was performed using factoMineR and factoextra R packages. Correlations between continuous variables were evaluated using Spearman’s rank test and were represented as correlation plots using Hmisc and corrplot R packages. In correlation plots, the size of the squares and circles is proportional to Spearman’s coefficient, the colour indicates if the correlation is positive or negative and asterisks indicate statistical significance. Differences were considered statistically significant when *p*-value < 0.05. Statistical analysis was performed using GraphPad Prism version 8.0 software and R software, version v4.1.1.

## Figures and Tables

**Table 1:** Characteristics of HD patients with and without active infection by SARS-CoV-2.

Characteristics	HD (N = 5)	HD + COVID-19 (N = 8)	*p*-Values
Age (Median, [IQR])	54.2 [(50–58)	55 (48–70)	0.5
Sex, male (n, %)	3 (60%)	6 (75%)	0.6
Active smoking (yes)	2 (40%)	2 (25%)	0.5
HD time, months (Median, IQR)	31 (31–36)	44 (29–47)	0.5
Use of EPO (n, %)	5 (100%)	5 (62.5%)	0.2
Previous kidney transplantation (n, %)	1 (20%)	1 (12.5%)	>0.9
Mean arterial pressure (Median, IQR)	103.7 (97.7–106)	88 (81.6–95.1)	0.1
Comorbidities (n, %)			
Obesity		1 (12.5%)	
Hypertension	5 (100%)	7 (87.5%)	0.5
Diabetes mellitus		3 (37.5%)	
Ischemic heart disease	2 (40%)	3 (37.5%)	>0.9
Dyslipidemia	2 (40%)	2 (25%)	>0.9
Cause of end-stage renal disease (n, %)			
Diabetic nephropathy		2 (25%)	
Hydronephrosis	2 (40%)	1 (12.5%)	0.5
Hypertensive nephrosclerosis		2 (25%)	
Hemolytic-uraemic syndrome	1 (20%)	1 (12.5%)	>0.9
IgA nephropathy	1 (20%)	2 (25%)	>0.9
Membranoproliferative glomerulonephritis	1 (20%)		
Laboratory parameters on test day (Median, [IQR])			
Serum creatinine, mg/dL	9.7 (6.2–10.1)	7.5 (5.5–9.4)	>0.9
C-reactive protein, mg/dL	0.3 (0.1–0.4)	3.6 (0.8–3.9)	*
Albumin, g/dL	4.3 (4.1–4.4)	3.5 (3.3–4.4)	0.1
ALT, U/L	10 (8–11)	16 (9–32.8)	0.2
AST, U/L	10 (10–11)	20.5 (16.3–26.8)	*
Platelets, 10^3^ cell/µL	125 (116.8–149.3)	183 (147.8–205.3)	0.2
Neutrophils, 10^3^ cell/µL	3.5 (3–4.4)	5.1 (4.1–5.5)	0.5
Lymphocytes, 10^3^ cell/µL	1.1 (1–1.3)	0.6 (0.4–1.1)	0.1
Monocytes, 10^3^ cell/µL	0.6 (0.4–0.7)	0.5 (0.3–0.6)	0.6
Fribrinogen, ng/mL	493 (406–495)	545 (481–662.8)	0.1
D-dimer, ng/mL	336 (247–660)	906 (467–1300)	0.4
IL6 ^a^, pg/mL	1.34 (0.2–2)	5.9 (1.93–13.9)	0.06
LDH, U/L	227.5 (215.8–239.2)	290 (252.5–306)	0.6
Saturation O2, % (Median, [IQR])		98 (98–100)	
Temperature, °C (Median, [IQR])		38 (36.5–38.6)	
COVID-19 treatment on test day (n, %)			
Hydroxychloroquine		1 (12.5%)	
Antibiotic (Amoxicillin/clavulanic acid, azithromycin, cephalosporin or carbapenem)		5 (62.5%)	
Corticosteroids		2 (25%)	
Tozilizumab		1 (12.5%)	
Anticoagulation		5 (62.5%)	
Death (n, %)		2 (25%)	
Days PSO on test day (Median, [IQR])		11 (6–14)	

^a^ Measured by Cytometric Bead Array (CBA, BD Bioscience, San Jose, CA, USA). ALT: Alanine aminotransferase; AST: aspartate aminotransferase; COVID-19: coronavirus disease 2019; EPO: erythropoietin; HD: hemodialysis; IQR: interquartile range; LDH: lactate dehydrogenase; PSO: post-symptom onset. *, *p* < 0.05.

## Data Availability

Data relating to the findings of this study are available from the corresponding author upon request.

## Supplementary Materials

The following supporting information can be downloaded at: https://www.mdpi.com/article/10.3390/ijms24021712/s1.
