# Supplementary material for: Hemodialysis-Associated Immune Dysregulation in SARS-CoV-2-Infected End-Stage Renal Disease Patients
Source: Int J Mol Sci. 2023 Jan 15;24(2):1712. doi: 10.3390/ijms24021712 (PMC9865754; doi:10.3390/ijms24021712)
Supplement: Supplementary file 1 [file ijms-24-01712-s001.zip › ijms-2125621-supplementary.pdf]

Supplementary tables:

Table S1. Characteristics of HD patients with COVID-19.

Patient	Age	Sex	Comorbidities	Cause of end-stage renal disease	HD time, month	Hospitalization[a]	Baseline Chest X-Ray[b]	PSO, days[c]	Worsening COVID-19 symptom after HD	Fever[d]	Diarrhea[d]	Asthenia[d]	Myalgia[d]	Dyspnea[d]	O_2 Saturation[e]	Exitus
P1	89	Male	Obesity, AH, DM, IHD Dyslipidemia	Diabetic nephropathy	25	YES	BI, AC	6	NO	YES	NO	YES	NO	NO	98	YES
P1*	89	Male	Obesity AH DM, IHD, Dyslipidemia	Diabetic nephropathy	25	YES	BI, AC	14	YES	NO	NO	YES	NO	YES	96	YES
P2	65	Male	AH, DM IHD, Dyslipidemia	Diabetic nephropathy	44	YES	GGO	3	NO	YES	NO	NO	NO	NO	98	YES
P3	48	Male	N/A	Hydronephrosis	53	NO	N/A	5	NO	NO	NO	NO	NO	NO	100	NO
P4	55	Female	AH, IHD	Hypertensive nephrosclerosis	44	YES	GGO, AC	9	NO	YES	NO	YES	YES	YES	95	NO
P5	52	Male	AH	IgA nephropathy	31	YES	N/A	46	YES	YES	YES	NO	YES	NO	98	NO
P6	34	Female	AH	Hemolytic-uraemic syndrome	45	YES	N/A	19	NO	YES	NO	NO	NO	NO	100	NO
P7	47	Male	AH, DM	IgA nephropathy	80	YES	GGO	13	YES	YES	NO	NO	NO	NO	100	NO
P8	84	Male	AH	Hypertensive nephrosclerosis	18	NO	N/A	11	NO	NO	NO	NO	NO	NO	100	NO

Two independent samples were obtained from P1 at different COVID-19 evolution time points (P1and P1); [a] Hospitalization due to COVID-19 disease; [b] Baseline Chest X-Ray at COVID-19 diagnosis; [c] Duration of symptoms from diagnosis by positive PCR to sample collection day; [d] Symptoms at debut of COVID-19 disease; [e] O_2 saturation measurement on sample collection day.

AC: Alveolar consolidation; AH: Arterial hypertension; BI: Bilateral infiltrates; DM: Diabetes mellitus; GGO: Ground-glass opacification; HD: hemodialysis; IHD: Ischemic heart disease; N/A: not applicable; PSO: Post-symptoms onset.

Table S2. Monoclonal antibodies for T subsets and monocytes analysis.

Marker	Fluorochrome	Vendor
CD3	PerCP-Cy5.5	BD Bioscience
CD4	APC-AlexaFluor750	Beckman Coulter
CD8	APC	BD Bioscience
HLA-DR	Pacific Blue	Beckman Coulter
CCR7	FITC	BD Bioscience
CD45RA	PCy7	BD Bioscience
CD3	V450	BD Bioscience
CD19	V450	BD Bioscience
CD56	V450	BD Bioscience
CD14	FITC	BD Bioscience
CD33	PCy7	eBioscience
HLA-DR	APC	BD Bioscience
CD16	V500	BD Bioscience
CCR2	PE	BD Bioscience
CCR5	APC-Cy7	BD Bioscience
CD86	PerCP-Cy5.5	BD Bioscience
BD Multitest 6-color TBNK reagent		BD Bioscience

Supplementary figures:

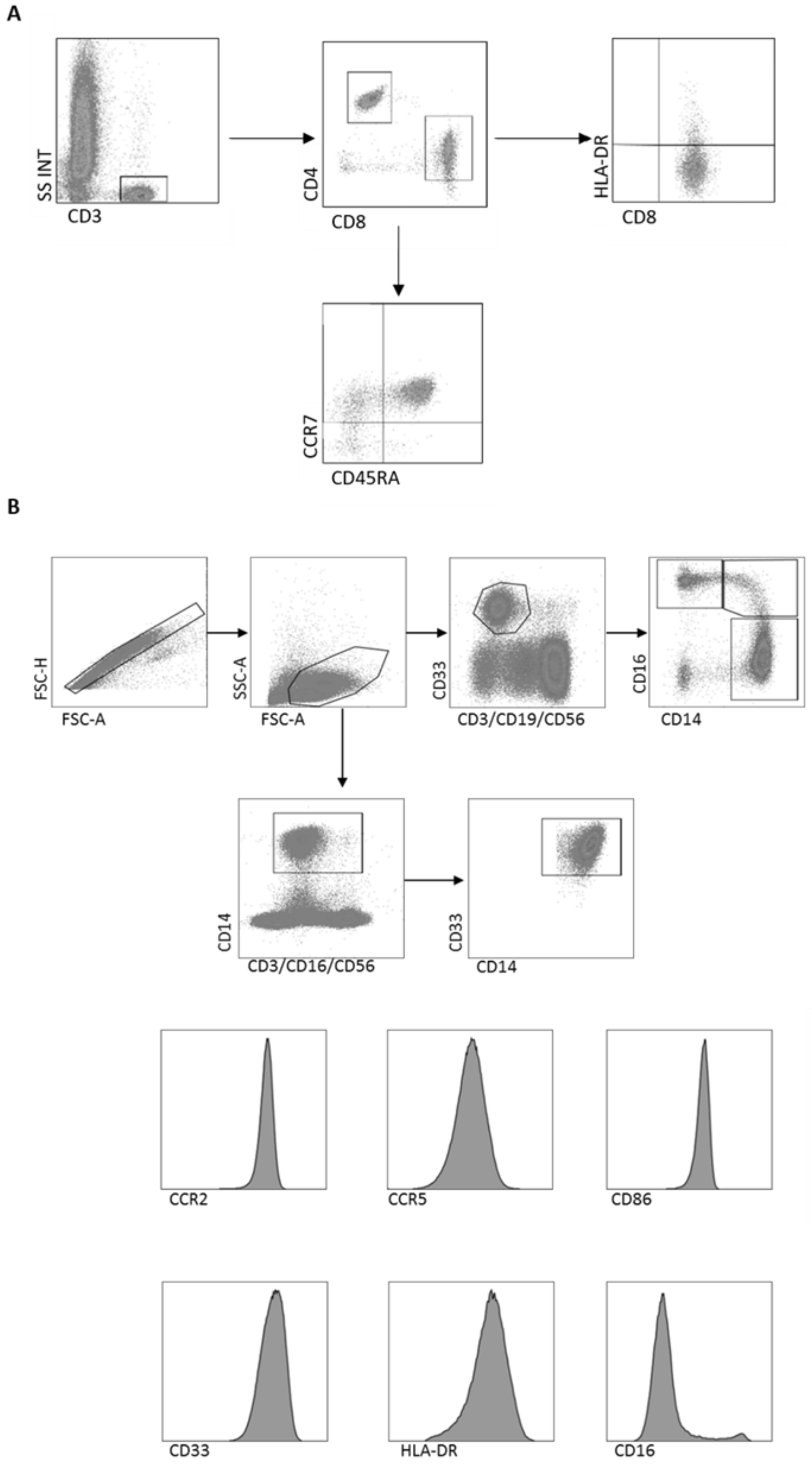

Figure S1. Gating strategy of T cells and monocytes subsets. (A) CD4^{+} and CD8^{+} T lymphocytes from gated total lymphocytes and their subsets naïve (CCR7^{+}CD45RA^{+}), CM (CCR7^{+}CD45RA^{-}), EM (CCR7^{-}CD45RA^{-}) and TEMRA (CCR7^{-}CD45RA^{+}). Activation (HLA-DR^{+}) from gated CD8^{+} T cells. (B) Monocytes subsets: classical (CD14^{+}CD16^{-}), intermediate (CD14^{+}CD16^{+}) and non-classical (CD14^{-}CD16^{+}) monocytes. Migration (CCR2, CCR5), co-stimulation (CD86) and antigen presentation (HLA-DR) markers gated from CD14^{+} cells.

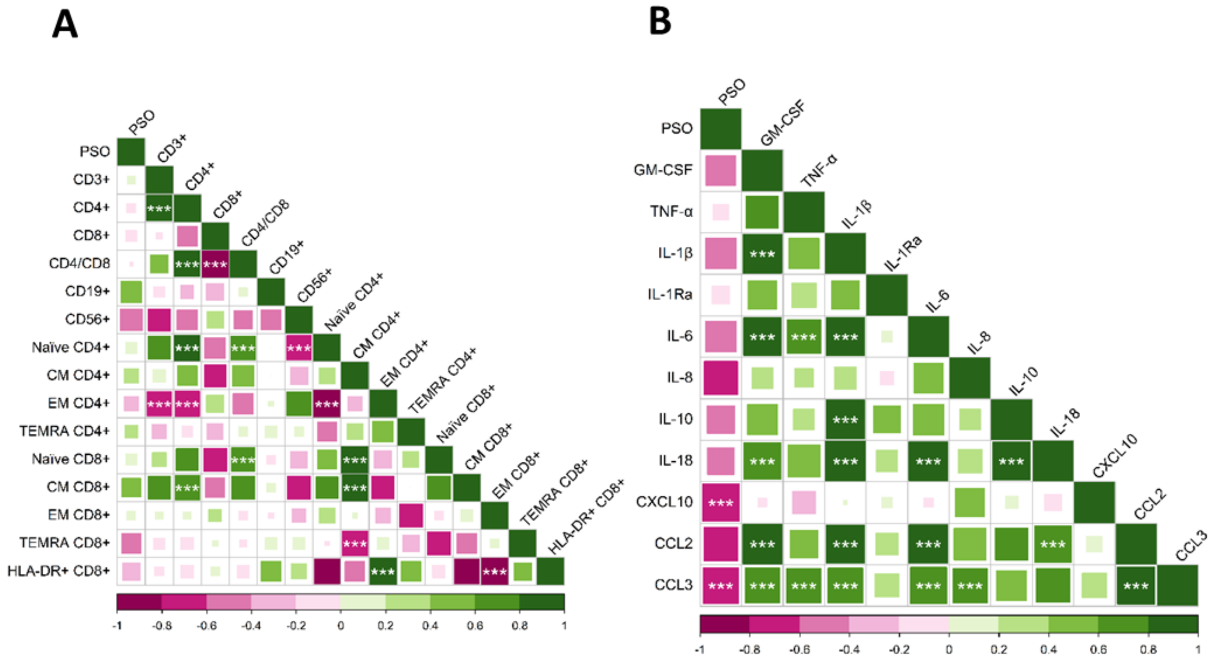

Figure S2. Correlation between cell populations or *in vitro* cytokines and days post symptom onset (PSO) in HD + COVID-19 patients. (A) Correlation plot between days PSO and subsets of CD4$^{+,}$ CD8^{+} T, B and NK cells in HD + COVID-19 patients. (B) Correlation plot between days PSO and cytokine levels produced by monocyte culture upon LPS stimulation in HD + COVID-19 patients. Positive correlations appear in green, and negative correlations appear in pink. The size and the color gradient of the circle corresponds to the magnitude of the correlation. Linear regressions were performed using Spearman's rank test, *, $p < 0.05$; **, $p < 0.01$; ***, $p < 0.001$.

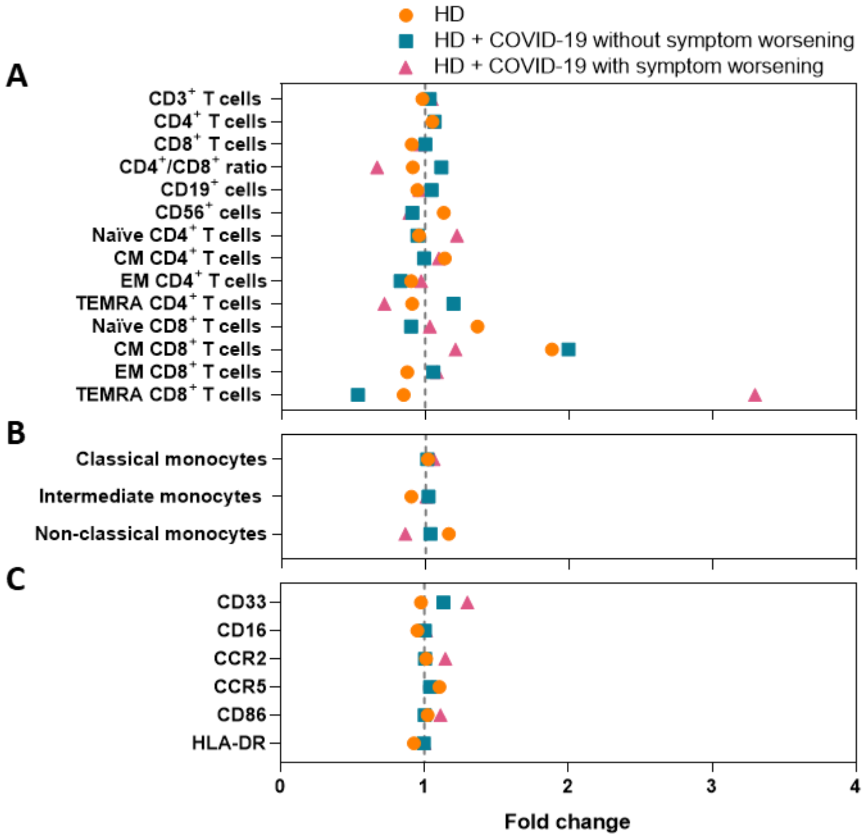

Figure S3. Change in lymphocyte and monocyte subsets after hemodialysis (A) Fold change of distribution of $CD4^+$ T, $CD8^+$ T, NK and B cells and their subsets (naïve, CM, EM and TEMRA) between uninfected HD patients, and HD + COVID-19 patients with or without clinical worsening after HD. (B) Fold change of classical ($CD14^+CD16^-$), intermediate ($CD14^+CD16^+$) and non-classical ($CD14^-CD16^+$) monocytes. (C) Fold change of surface markers on $CD14^+$ monocytes. Mean fold change is represented for each patient cohort. Dotted line indicates no change between the pre and post HD sample. All p values are non-significant, ns.
